# The crosstalk of NAD, ROS and autophagy in cellular health and ageing

**DOI:** 10.1007/s10522-020-09864-0

**Published:** 2020-03-03

**Authors:** Lucia Sedlackova, Viktor I. Korolchuk

**Affiliations:** Biosciences Institute, Faculty of Medical Sciences, Newcastle University, Campus for Ageing and Vitality, Newcastle upon Tyne, NE4 5PL UK

**Keywords:** NAD, ROS, Autophagy, Sirtuins, Acetylation, Ageing

## Abstract

Cellular adaptation to various types of stress requires a complex network of steps that altogether lead to reconstitution of redox balance, degradation of damaged macromolecules and restoration of cellular metabolism. Advances in our understanding of the interplay between cellular signalling and signal translation paint a complex picture of multi-layered paths of regulation. In this review we explore the link between cellular adaptation to metabolic and oxidative stresses by activation of autophagy, a crucial cellular catabolic pathway. Metabolic stress can lead to changes in the redox state of nicotinamide adenine dinucleotide (NAD), a co-factor in a variety of enzymatic reactions and thus trigger autophagy that acts to sequester intracellular components for recycling to support cellular growth. Likewise, autophagy is activated by oxidative stress to selectively recycle damaged macromolecules and organelles and thus maintain cellular viability. Multiple proteins that help regulate or execute autophagy are targets of post-translational modifications (PTMs) that have an effect on their localization, binding affinity or enzymatic activity. These PTMs include acetylation, a reversible enzymatic modification of a protein’s lysine residues, and oxidation, a set of reversible and irreversible modifications by free radicals. Here we highlight the latest findings and outstanding questions on the interplay of autophagy with metabolic stress, presenting as changes in NAD levels, and oxidative stress, with a focus on autophagy proteins that are regulated by both, oxidation and acetylation. We further explore the relevance of this multi-layered signalling to healthy human ageing and their potential role in human disease.

## Introduction

NAD depletion, oxidative stress and loss of macroautophagy (from herein referred to as autophagy) efficiency have all been linked to healthy, pathological and premature ageing (Kubben and Misteli 2017; López-Otín et al. 2013, 2016). Individually, these alterations may underlie seven of the nine outlined hallmarks of ageing including genomic instability (all), telomere attrition (oxidative stress), epigenetic alterations (NAD), loss of proteostasis (autophagy), de-regulated nutrient sensing (NAD), cellular senescence (all) and mitochondrial dysfunction (all) (López-Otín et al. 2013, 2016). Moreover, it is becoming increasingly clear that a significant degree of crossover and interdependence between the three phenomena occur in ageing cells and tissues. Specifically, increased reactive oxygen species (ROS) and depletion of NAD can impact autophagy by influencing post-translational modifications (PTMs) of autophagy proteins (Filomeni et al. 2015; Sedlackova et al. 2020; Zhang et al. 2016a). Furthermore, autophagy impairment may lead to the failure to reconstitute cellular metabolism and detoxify oxidised substrates (Li et al. 2015; Morishita and Mizushima 2019).

### Nicotinamide adenine dinucleotide (NAD)

**Fig. 1:**
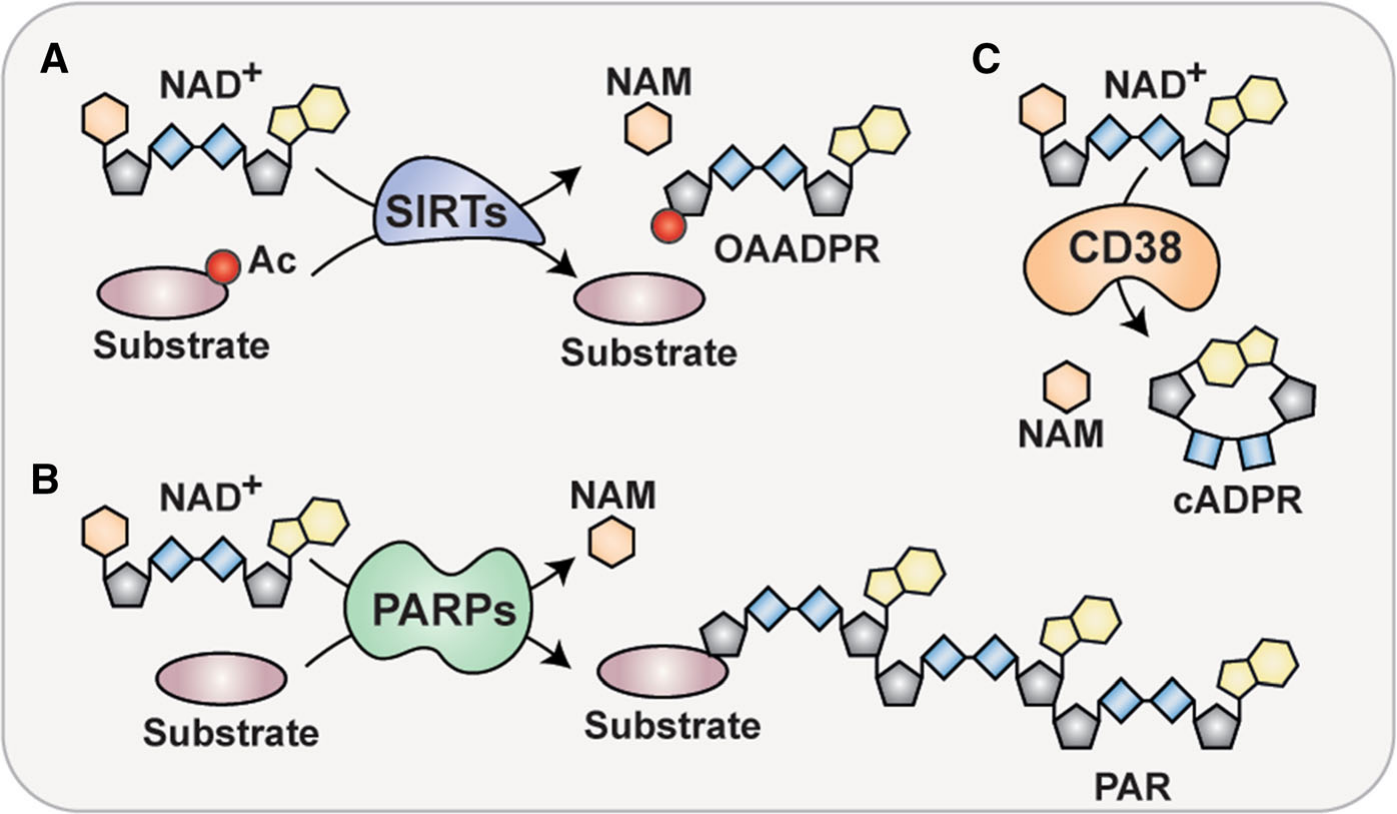
Molecular outcomes of NAD^+^ cleavage. The three major groups of NAD^+^-consuming enzymes include sirtuins (SIRT), poly(ADP-ribose) polymerases (PARPs) and cyclic ADP-ribose synthases (cADPRs, CD38, CD157). **a** SIRT1-3 are NAD + -dependent deacetylases that bind an acetylated (Ac) protein substrates and transfer the Ac moiety onto ADP-ribose (ADPR) to give rise to *O*-acetyl-ADP-ribose, a deacetylated protein substrate and a by-product of the reaction, nicotinamide (NAM). **b** PARPs are indiscriminate NAD^+^ consumers that use NAD^+^ as a co-substrate to generate poly(ADP)-ribose (PAR) chains on protein substrates, and generate NAM as a by-product. **c** cADPRs consume NAD^+^ to generate cyclic ADP-ribose (cADPR), a second messenger, and a by-product, NAM

NAD is an essential metabolite that participates in cellular energy generation and signalling. When plentiful, the redox balance and availability of NAD aid cellular adaptation to metabolic stress and help maintain genomic stability, mitochondrial function, detoxification of ROS and cell survival (Fang et al. 2017). Due to its ability to accept or donate electrons, NAD in its reduced (NADH) or oxidised (NAD^+^) form assists energy metabolism in the cytosol and within mitochondria (Canto et al. 2015). In addition, NAD^+^ is cleaved into ADP-ribose (ADPR) and nicotinamide (NAM) by three classes of enzymes: sirtuins (SIRTs), poly(ADPR) polymerases (PARPs) and cyclic ADPR synthases (CD38 and CD157) (Fig. 1), which require ADPR for their enzymatic activity (Canto et al. 2015; Fang et al. 2017). Crucially, although SIRT activity depends on NAD^+^ availability and cannot contribute to uncontrolled NAD^+^ cleavage, PARPs and CD38 are known for their indiscriminate NAD^+^ consumption and their role in age- and disease-related NAD depletion (Canto et al. 2015). Homeostasis of intracellular NAD pools is maintained by either local synthesis from NAD^+^ precursors (nicotinamide (NAM), nicotinamide riboside (NR) or nicotinamide mononucleotide (NMN)) or centralised de novo synthesis from nicotinic acid or L-tryptophan (Canto et al. 2015). Therefore, it is the balance between NAD^+^ cleavage and synthesis that dictates the total intracellular NAD pool, and by extension, cellular metabolism and protein acetylation status (Strømland et al. 2019).

### Reactive oxygen species (ROS)

ROS are highly reactive molecules of oxygen, which harbour one unpaired electron (superoxide anion (O_2_^•−^), hydroxyl radical (OH^•^)) or an additional electron pair (H_2_O_2_) on its valence orbital (Halliwell and Gutteridge 2015). The increased electron content in oxygen molecules makes them more reactive and more likely to participate in one-electron oxidative transfer reactions that lead to macromolecule modification and/or damage (Halliwell and Gutteridge 2015). ROS can also interact with nitric oxide to generate reactive nitrogen species (RNS), including peroxynitrite (ONOO^−^) (Bartesaghi and Radi 2018). Electrons from ROS/RNS interact with amino acid residues incorporated in proteins and thus translate the cellular redox state into protein activating or inhibiting signals by the modulation of protein enzymatic activity, binding affinity or structural conformation. Particularly sensitive to ROS-mediated redox regulation are cysteine (Cys) residues (Bischoff and Schlüter 2012). Cys is one of the least represented, and yet often highly conserved amino acids that participates in protein structural integrity by formation of covalent disulphide-bridges between two cysteine residues, or protein enzymatic activity, i.e. by thioester bond formation or co-factor stabilisation (Bak et al. 2019; Marino and Gladyshev 2010).

### Autophagy

Autophagy is a cytosolic pathway of dynamic membrane rearrangement and cargo sequestration that is assisted and executed by a set of highly conserved autophagy (ATG) proteins (Dikic and Elazar 2018). Autophagy is a catabolic process responsible for cargo recognition, its engulfment in a double membraned vesicle called autophagosome and delivery to the lysosomal lumen for degradation. The subsequent release of amino acids, lipids and nucleosides reconstitutes cellular homeostasis and sustains viability in times of stress (Morishita and Mizushima 2019). The molecular execution of autophagy initiation is mediated by ATG protein association into functional complexes known as the Unc-51-like kinase 1 (ULK1) complex, the class III phosphatidylinositol 3 kinase (PI(3)K) complex, the ATG9-membrane complex, an ATG2–WIPI (WD-repeat protein interacting with phosphoinositides) complex and two conjugation systems consisting of the ATG3-ATG8/LC3 and the ATG5-ATG12:ATG16L complex (Table 1) (Suzuki et al. 2017). The combined action of these complexes is responsible for ER localization of all autophagy components and for the formation and maturation of the autophagic membrane. In addition, a group of autophagy receptors, e.g. sequestosome 1 (SQSTM1/p62), is then responsible for spatially linking the ubiquitylated cargo, including long-lived or aggregated proteins, pathogens and organelles, to the growing autophagosome (Dikic and Elazar 2018; Johansen and Lamark 2019).

**Table 1:** Acetylation-sensitive proteins in autophagy

Protein	Function in autophagy	Acetylation sensitive Lys residues	Acetylase	Deacetylase	Outcome of deacetylation
TFEB	Transcription factor	(Lys^91^), (Lys^103^), Lys^116^, Lys^274^, Lys^279^ and (Lys^430^)	ACAT1? GCN5	SIRT1 HDAC2/6	Increased lysosomal biogenesis, transcription of ATG proteins
FoxO1	Transcription factor	Lys^242^, Lys^245^, Lys^262 (mouse residues)^	CBP,p300	SIRT1	Increased DNA binding
FoxO3a	Transcription factor	Unknown	Unknown	SIRT1-3	Cytoplasm to nucleus translocation
ULK1	ULK1 complex member	Lys^162^ and Lys^606^	TIP60	Unknown	Loss of kinase activity stimulation
VPS34	Class III PI3K kinase complex member	Lys^29^, Lys^771^, (Lys^781^)	p300	Unknown	Increased complex formation (Lys^29^), increased PI binding (Lys^771^)
Beclin 1	Class III PI3K kinase complex member	Lys^430^ and Lys^437^	p300	SIRT1	Autophagosome maturation
ATG3	Autophagosome elongation	Lys19, Lys48, Lys183 (yeast residues)	TIP60	HDAC1/2	Decreased membrane-binding
ATG7	Autophagosome elongation	Unknown	p300	SIRT1	Increased LC3-PE formation
ATG5	LC3–PE deconjugation	Unknown	p300	SIRT1 SIRT2 SIRT3	Increased LC3-PE formation
ATG12	LC3–PE deconjugation	Unknown	p300	Unknown	Increased LC3-PE formation
LC3	Multiple	Lys^49^ and Lys^51^	p300	SIRT1	Increased levels of LC3-PE formation
Ub	Selectivity	Lys^6^ and Lys^48^	Unknown	Unknown	Poly-Ub chain formation
p300	Inhibits autophagy	Multiple	p300	SIRT2	Loss of inhibitory ATG5/ATG7/ATG12/LC3 acetylation
p62	Selective cargo recognition	Lys^420^ and Lys^435^	TIP60	HDAC6	Increased Ub binding

() lysine residues sensitive to acetylation, but their involvement in autophagy regulation remains unknown

The canonical pathway of starvation-induced autophagy was long thought to rely on phosphorylation cascades that are triggered by the loss of nutrient signalling and converge on a small number of regulating kinase complexes (Beurel et al. 2015; Rabanal-Ruiz et al. 2017; Tamargo-Gómez and Mariño 2018). These regulators then either lose function and thus release downstream autophagy components from an inhibitory state, or become activated and promote autophagy initiation. In addition, multiple layers of regulation involved in autophagy initiation, cargo sequestration and degradation, incorporate various stress signals and often improve the efficiency of autophagic flux via PTMs of autophagy proteins or their upstream regulators (Filomeni et al. 2015; Montagna et al. 2016; Sedlackova et al. 2020; Zhang et al. 2016a).

In this review, we explore the current knowledge of how two types of PTMs, lysine (Lys) acetylation and cysteine (Cys) oxidation, regulate the abundance and activity of ATG proteins, and highlight which Lys modifications are subject to NAD^+^ availability. We then summarize the main concepts of autophagy regulation by oxidative stress and discuss the implications and consequences of age-related changes to NAD^+^ availability and an increase in oxidative stress on the efficiency of autophagy. We further explore whether autophagy directly influences the homeostasis of cellular NAD levels and outline how aberrations in either of the three phenomena could lead to dysfunction observed in physiological and pathological ageing.

## Targets of acetylation in autophagy

Lysine acetylation is a major reversible PTM in eukaryotes that arises by donation of the acetyl moiety from acetyl coenzyme A (Ac-CoA) via its re-direction from mitochondrial energy generation (Drazic et al. 2016). Protein acetylation status is balanced by the activity of multiple lysine acetyl transferases (KATs, historically known as histone acetyl transferases HATs) and lysine deacetylases (KDAC, or HDACs) (Narita et al. 2019). KATs catalyse acetyl moiety transfer from Ac–CoA to a lysine residue of the target protein, while KDACs cleave and release the acetyl moiety (KDAC, classes I, II and IV) or catalyse transfer of the acetyl moiety onto ADPR, a product of NAD^+^ cleavage (class III KDACs, sirtuins (SIRTs) (Fig. 1). Acetylation status of autophagy proteins is largely controlled by p300 (KAT3B) and 60 kDa Tat-interactive protein (TIP60/KAT5) KATs and SIRT1-3 and HDAC2/6 KDACs (summarized in Table 1). In the next section, we explore how protein acetylation status, generally high in conditions of nutrient abundance and low under nutrient starvation, regulates the activity and localisation of TFs, proteins and receptors involved in autophagy.

### Regulation of transcription factors involved in autophagy gene transcription

**Fig. 2:**
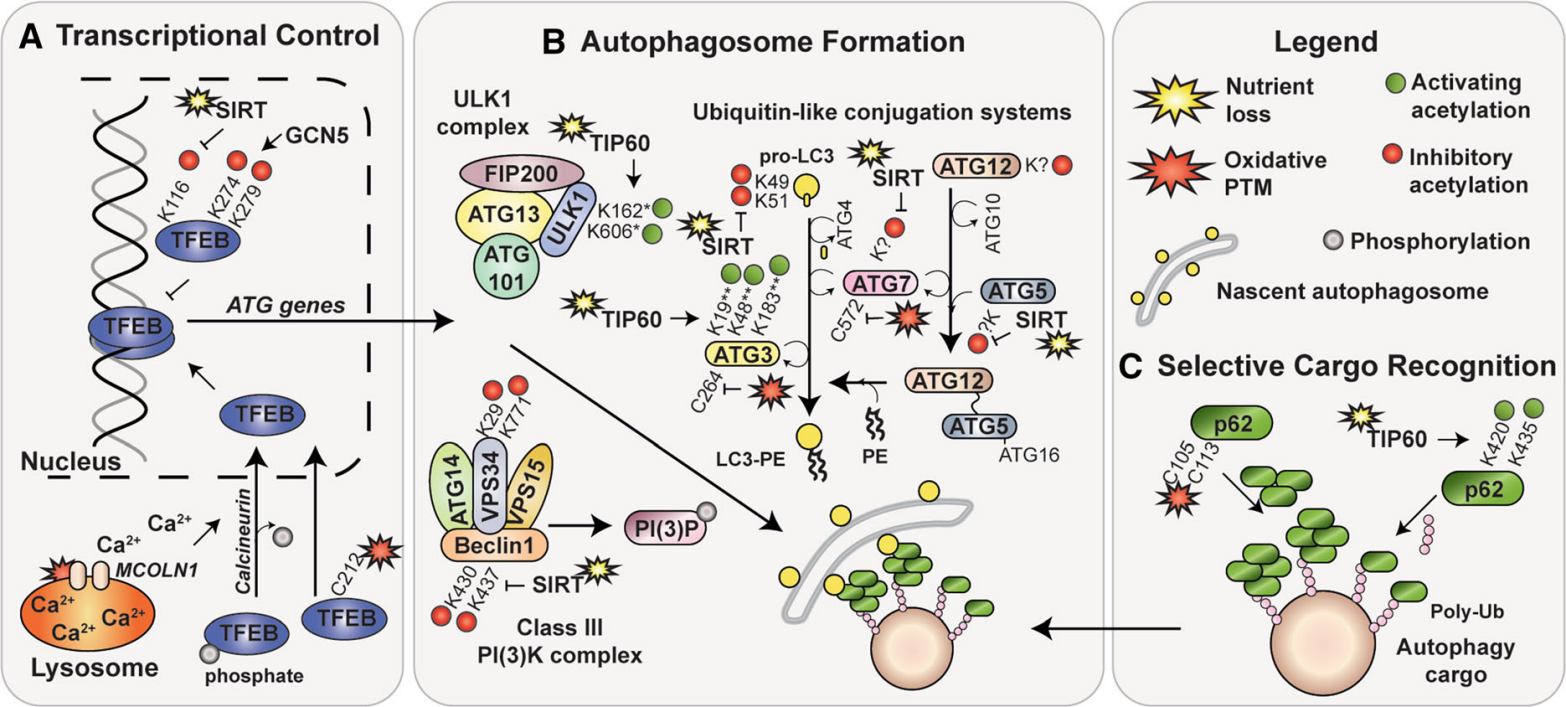
Autophagy targets of acetylation and oxidation. Nutrient and oxidative stresses affect proteins that participate in autophagy by lysine (K) acetylation or cysteine (C) oxidation. **a** Localization of transcriptional factor EB (TFEB), a master regulator of autophagy and lysosomal gene expression, is regulated by oxidative stress. Indirectly, oxidative modification of mucolipin 1 (MCOLN1) leads to TFEB dephosphorylation by Ca^2+^-sensitive phosphatase, calcineurin and its translocation to the nucleus. Directly, oxidation of TFEBs redox-sensitive residue, C^212^, promotes rapid nuclear localization. In addition, inhibitory lysine acetylation of K^274^ and K^279^ that is regulated by the general control non-repressed protein 5 (GCN5) prevents TFEB dimerization. The molecular and functional outcomes of K^116^ are not known, but are opposed by nutrient sensitive, NAD + -dependent lysine deacetylase (KDAC), SIRT1. **b** Acetylation-sensitive lysine residues were detected within members of the ULK1 complex, the class III PI(3)K complex and both ubiquitin-like conjugation systems. Unc-51-like kinase 1 (ULK1, ULK1 complex) contains two lysine residues, K^162^ and K^606^ (* in mouse) that are acetylated by TIP60 in response to serum starvation. Vacuolar protein sorting 34 (VPS34, class III PI(3)K complex) contains two acetylation sensitive lysine residues (K^29^ and K^771^) that are subject to inhibitory acetylation in fed conditions. Inhibitory acetylation of residues K^430^ and K^437^ in Beclin 1 (class III PI(3)K complex) is opposed by nutrient-sensitive SIRT1 KDAC. Within the ubiquitin-like conjugation systems, LC3 (K^49^, K^51^), ATG5 (unknown), ATG7 (unknown) and ATG12 (unknown) are subject to inhibitory acetylation by p300 (not shown) in fed conditions. Acetylation of LC3, ATG5 and ATG7 residues is opposed by SIRT1 deacetylase. ATG3 is subject to activating acetylation by TIP60 in starved conditions. Acetylation of lysine residues K^19^, K^48^ and K^183^ (** in yeast) is necessary for ATG3 enzymatic activity and LC3 binding affinity. ATG3 and ATG7 are also subject to inactivation by oxidative stress due to oxidation of their catalytic thiols, C^264^ and C^572^, respectively. **c** Selective autophagy receptor p62 is a target of both, acetylation and oxidation. TIP60-dependent activating acetylation of K^420^ and K^435^ residues within the ubiquitin-associated (UBA) domain prevent UBA dimerization and enhance ubiquitin (Ub) binding affinity. Oxidation of C^105^ and C^113^ promotes p62 oligomerization and stimulates autophagy by intermolecular disulphide bond formation

The loss of lysine acetylation triggers stimulation of several TFs involved in the transcription of *ATG* genes (Fig. 2a) (Füllgrabe et al. 2016). The strongest link between TF deacetylation and autophagy stimulation comes from studies of transcription factor EB (TFEB), a member of the microphthalmia family of bHLH-LZ transcription factors (Mit/TFE), a group of TFs that stimulate lysosomal biogenesis and expression of autophagy proteins (Yang et al. 2018). Specifically, TFEB is responsible for transcription of multiple autophagy genes (*ATG4, ATG9B, MAP1LC3B (*LC3B*), UVRAG* (UV radiation resistance associated gene)*, WIPI* (WD repeat domain phosphoinositide-interacting protein 1), and *SQSTM1* (p62)) (Füllgrabe et al. 2016; Settembre et al. 2011). Acetylation of a conserved lysine residue Lys^116^ was independently identified in three studies as a modifier of TFEB activity in microglia (Bao et al. 2016) and in cancer cells (Wang et al. 2019b; Zhang et al. 2018). In microglia, Lys^116^ was directly deacetylated by SIRT1 which promoted degradation of fibrillar amyloid β (Bao et al. 2016). In cultured cells, treatment with a KDAC inhibitor, suberoylanilide hydroxamic acid (SAHA), increased the transcriptional activity of TFEB and influenced acetylation of four lysine residues (Lys^91^, Lys^103^, Lys^116^ and Lys^430^) (Zhang et al. 2018). In addition, authors of this study identified acetyl-coenzyme A acetyltransferase 1 (ACAT1) and HDAC2 as modulators of the overall TFEB acetylation status. Furthermore, a study in a model of chronic kidney disease identified HDAC6 as another KDAC involved in the regulation of TFEB activity (Brijmohan et al. 2018). Importantly, authors of neither of the studies demonstrated a direct interaction between TFEB and HDAC2 or HDAC6, respectively (Brijmohan et al. 2018; Zhang et al. 2018). Overexpression of another KAT, the general control non-repressed protein 5 (GCN5/KAT2A), but not TIP60, p300 or CREB-binding protein (CBP), led to increased TFEB acetylation of Lys^116^, Lys^274^ and Lys^279^ residues (Wang et al. 2019b). Authors further demonstrated that TFEB acetylation at Lys^274^ and Lys^279^ mechanistically disrupts TFEB dimerization and its ability to bind DNA, and thus negatively regulates expression of lysosomal and autophagy genes (Fig. 2A) (Wang et al. 2019b). Crucially, Lys^116^ of TFEB is not conserved in *Drosophila melanogaster* and *Caenorhabditis elegans* or in other members of the Mit-TFE family (Wang et al. 2019b), thus SIRT1 and HDAC regulation of TFEB activity is likely to be unique to vertebrates.

Additionally, two members of the forkhead box class O (FoxO) TF family, FoxO1 and FoxO3a, recognized for their role in autophagy/mitophagy (*ATG4, ATG5, ATG12, ATG14, BECN1* (beclin 1)*, BNIP3* (BCL2 interacting protein 3)*, LC3B, ULK1, VPS34* (vacuolar protein sorting 34) *GABARAPL1* (gamma-aminobutyric acid receptor-associated protein-like1), and *PARK6/PINK1* (PTEN-induced kinase 1)) gene transcription, are regulated by acetylation PTMs (Fang et al. 2019; Füllgrabe et al. 2016; Requejo-Aguilar et al. 2015). It was first demonstrated that FoxO1 acetylation on Lys^242^, Lys^245^ and Lys^262^ residues (in mice) by CBP is opposed by SIRT1 in response to serum (Daitoku et al. 2004) and glucose starvation (Hariharan et al. 2010). Mechanistically, acetylation of the three Lys residues within FoxO1 interferes with its DNA binding and inhibits its transcriptional activity (Matsuzaki et al. 2005). Furthermore, FoxO1 acetylation permits access for upstream kinases to phosphorylate its Ser^253^ residue that is otherwise shielded by FoxO1-DNA complex formation (Matsuzaki et al. 2005). FoxO1 phosphorylation sites have since became known to act as docking or shielding sites for 14–3-3 protein binding, and the heterodimer exit into and retention within the cytoplasm (Brunet et al. 1999; Saline et al. 2019).

Similarly to FoxO1, the transcriptional activity of FoxO3 is modulated by SIRT1-3 deacetylases, though the Lys residues susceptible to acetylation remain unknown. First, caloric restriction and oxidative stress increase SIRT2 expression, decrease FoxO3 acetylation and improve gene transcription (Wang et al. 2007). In mitochondria, SIRT3 mediated deacetylation of the mitochondrial FoxO3 pool and led to cellular detoxification of oxidative stress by increased expression of the mitochondrial superoxide dismutase (Jacobs et al. 2008). However, some controversy exists in the perceived outcome of FoxO acetylation. A few articles published in early 2000s reported entirely opposite findings, demonstrating that FoxO acetylation improves transcription of its target genes (Motta et al. 2004; Yang et al. 2005). The pitfall of the majority of FoxO studies centres on the lack of distinction between CBP-mediated acetylation of FoxO, which may increase its DNA binding, and acetylation of histones, which would relax the chromatin condensation and promote gene transcription (discussed in detail in (Daitoku et al. 2011)).

Overall, transcriptional activity of TFEB, a member of the Mit/TFE family, and two FoxO isoforms, FoxO1 and FoxO3, is regulated by acetylation. Three classes of KDACs, SIRT1, HDAC2/6 and GCN5, are thought to deacetylate multiple and variable Lys residues in TFEB, of which the SIRT1 target, Lys^116^, is unique to vertebrates, and Lys^274^ and Lys^279^ deacetylation regulates TFEB-DNA complex formation (Wang et al. 2019b). In the FoxO family, FoxO1 is the better studied isoform, with known target Lys residues in the mouse (Table 1), though both FoxO1 and FoxO3a are likely activated by SIRT-mediated deacetylation. Thus, transcriptional regulation of autophagy/mitophagy genes by TFEB and FoxO1/3a is, at least in part, responsive to intracellular NAD^+^ levels that influence SIRT activity.

### Regulation of autophagy protein complexes

Several autophagy proteins involved in autophagosome formation, growth and maturation may be modified by acetylation (Table 1). Studies from the last decade, summarized below, identify lysine residues sensitive to acetylation in members of the ULK1 kinase complex, the class III PI(3)K kinase complex and the two conjugation systems, ATG12 (ATG7, ATG10, ATG5) and LC3 (ATG4, ATG7, ATG3), as well as ATG12 and LC3 themselves (Fig. 2B).

In the ULK1 complex, ULK1 itself is a target of acetylation by TIP60 (Lin et al. 2012). In serum starved cells, ULK1 was shown to be a target of GSK3-dependent and TIP60-mediated acetylation of two crucial residues, Lys^162^ and Lys^606^ (in mouse; likely Lys^162^ and Lys^607^ in human), that together stimulate its kinase activity and promote autophagy initiation (Lin et al. 2012). Furthermore, oxidative stress that induces ER stress, was also shown to stimulate ULK1 acetylation by a GSK3-TIP60-dependent mechanism (Nie et al. 2016). These studies together support the idea that ULK1 kinase activity can be modulated by oxidative and metabolic stress via an upstream signalling cascade that results in TIP60 activation and ULK1 acetylation.

Within the Class III PI(3)K complex, VPS34 kinase acetylation by p300 occurs on residues Lys^29^, Lys^771^ and Lys^781^ and inhibits its lipid kinase activity and PI(3)P production (Su et al. 2017). It was further determined that acetylation of Lys^29^ residue prevents VPS34 association with Beclin 1 that is required for the formation of a complex involved in autophagy progression. Another layer of VPS34 activity regulation occurs upon acetylation of the Lys^771^ residue located within its catalytic site. In a manner similar to the level of regulation at the Lys^29^ residue, acetylation of Lys^771^ disrupts binding between VPS34 and its substrate, PI (Su et al. 2017). However, the KDAC responsible for Lys^29^ and Lys^771^ deacetylation remains unknown. In addition to VPS34, Beclin 1 of the Class III PI(3)K complex is also a target of inhibitory acetylation on Lys^430^ and Lys^437^ residues by p300 (Sun et al. 2015). Beclin 1 acetylation was demonstrated to promote its binding to Rubicon, and thus shown to result in the loss of autophagosome maturation (Ohashi et al. 2019; Sun et al. 2015). Furthermore, in vitro acetylation analysis revealed that SIRT1 is preferentially responsible for Beclin 1 deacetylation (Sun et al. 2015).

Next, SIRT1-mediated deacetylation of nuclear LC3 at Lys^49^ and Lys^51^ residues initiates LC3 translocation to the cytoplasm via a diabetes and obesity regulated (DOR/TP53INP2)-dependent interaction with deacetylated LC3 (Huang et al. 2015). DOR then further assists in LC3 localization to nascent autophagosomes thanks to its ATG7-binding affinity (You et al. 2019b). Furthermore, DOR also contains a ubiquitin-interacting motif and is thus likely to promote LC3-ATG7 formation in the vicinity of ubiquitylated cargo (Xu and Wan 2019; You et al. 2019b). Upon relocation to the cytoplasm, LC3 Lys^49^ and Lys^51^ acetylation, that is lost upon nutrient starvation, was recently shown to completely abolish p62 binding (Song et al. 2019). Due to the location and conservation of the two critical lysine residues in the hydrophobic binding grooves of LC3 (Huang et al. 2015; Song et al. 2019), it stands to reason that Lys^49^ and Lys^51^ acetylation could disrupt LC3 interaction with multiple binding partners including, but not limited to DOR and p62. Altogether, LC3 deacetylation in response to nutrient starvation not only promotes its exit from the nucleus, but also determines substrate binding specificity of protein partners via their LC3-interacting regions (LIRs).

Cytoplasmic LC3 targeting to and docking on the nascent autophagosomes requires covalent conjugation of LC3 to phosphatidylethanolamine (PE). In a ubiquitin-like conjugation system, ATG7 (and E1-like enzyme), ATG3 (an E2-like enzyme) and an ATG5-ATG12:ATG16L complex (an E3-like enzyme) assist LC3 conjugation to PE (Dikic and Elazar 2018). Nutrient starvation in yeast was first reported to decrease or not change acetylation levels of ATG proteins, with the notable exception of ATG3, in which Lys^19^, Lys^48^ and Lys^183^ acetylation increased (Yi et al. 2012). Authors of this study had further shown that while acetylation of Lys^183^ is crucial for the enzymatic activity of ATG3, Lys^19^ and Lys^48^ acetylation was crucial for autophagy progression by improving interaction between ATG3 and ATG8 (LC3 in mammals), and was regulated by the opposing activities of the yeast histone acetyltransferase Esa1 (TIP60/KAT5 orthologue)) and a histone deacetylase Rpd3 (HDAC1/2 orthologue) enzymes. Furthermore, ATG3 acetylation on Lys^19^ and Lys^48^ was shown to enhance its ER membrane localization and binding in vitro (Li et al. 2017).

Other members of the ubiquitin-like conjugation system, ATG7, ATG5 and ATG12, are targets of p300-mediated acetylation (Lee and Finkel 2009) and SIRT1-dependent deacetylation (Lee et al. 2008). In direct contrast to ULK1 and ATG3, acetylation of these ATG proteins generally inhibits their function. However, the specific residues, their location and effect of acetylation on the structure or function of ATG proteins remains unknown. Structural studies of the ATG12-ATG5:ATG16 complex (Otomo et al. 2013) and the nature of interaction between ATG12 and ATG3 (Metlagel et al. 2013) point towards several key lysine residues that could be targets of acetylation in ATG12. First, lysine residues 60, 69, 71 and 128 located on the surface of ATG12 (Metlagel et al. 2013) could contribute to binding affinity between ATG12 (E3-like) and ATG3 (E2-like) that is required for the spatiotemporal regulation of LC3 lipidation. Furthermore, ATG5 contains multiple lysine residues, of which Lys^53^, Lys^130^, Lys^171^ are conserved (Matsushita et al. 2007). Although Lys^130^ is the known catalytic site for conjugation between ATG5 and ATG12 (Mizushima et al. 1998), the function and acetylation-sensitivity of Lys^53^ and Lys^171^ remain unknown. Lastly, no published study followed-up reports of ATG7 acetylation-sensitivity (Lee et al. 2008; Lee and Finkel 2009). However, a high resolution mass spectrometry study of global protein acetylation identified Lys^306^ of the human ATG7 protein as a residue that might be relevant for further study (Choudhary et al. 2009). Thus, although ATG5, ATG7 and ATG12 have been known substrates of p300 and SIRT for almost a decade, the lysine residues sensitive to acetylation, or indeed the nature of protein inhibition by acetylation have not been elucidated.

### Selective cargo recognition

Autophagy receptors modulate the selectivity and specificity of cargo recognition in the autophagy pathway. Although the current knowledge of about PTMs that affect the structure, function and localisation of the canonical autophagy receptors is fairly limited, phosphorylation and ubiquitylation sites were identified in all canonical receptors (THANATOS, https://thanatos.biocuckoo.org) (Deng et al. 2018). The best characterization of acetylation-dependent regulation of autophagy receptors concerns the p62 protein and its affinity for ubiquitin (Fig. 2c). Binding between ubiquitin and p62 to spatially link cargo to the forming autophagosome is, in fed condition, restricted due to the low binding activity of the ubiquitin associated (UBA) domain of p62 and further restricted by UBA homodimerisation (Long et al. 2010). Briefly, Lys^420^ monoubiquitylation (Lee et al. 2017; Peng et al. 2017), and Ser^403^ and Ser^407^ (in humans; Ser^405^ and Ser^409^ in mice) phosphorylation (Matsumoto et al. 2015) strengthen the interaction and binding affinity between p62 and ubiquitin. In addition, acetylation of p62 Lys^420^ and Lys^435^ residues, regulated by TIP60 and opposed by HDAC6 upon serum and amino acid starvation, interferes with UBA dimerization (Lys^420^ and Lys^435^) and enhances ubiquitin-binding affinity (Lys^435^) (You et al. 2019a). Moreover, spatial proximity between p62 and HDAC6 at sites of protein aggregation promotes their interaction and regulation of HDAC6 deacetylase activity and, by extension, protein aggregate recycling by p62 (Yan et al. 2013).

Moreover, ubiquitin (Ub) itself is a target of lysine acetylation (Ohtake et al. 2015). Formation of stable polyubiquitin chains by covalent linkages of single Ub moieties via homotypic Lys^63^ linkage (also known as K63) promotes autophagy (Grumati and Dikic 2018). Although the KAT(s) and KDAC(s) involved and the physiological relevance of Ub acetylation remain unknown, acetylation of Lys^6^ and Lys^48^ residues was shown to interfere with poly-Ub chain formation (Lys^11^-, Lys^48^- and Lys^63^-linked) in vitro (Ohtake et al. 2015).

### Demystification of the acetylation riddle in autophagy regulation

Recent advances in our understanding of which KATs and KDACs are involved in the regulation of autophagy protein acetylation highlight a few interesting phenomena. Overall, autophagy protein acetylation status is mainly regulated by p300, CREB binding protein (CBP) and TIP60 KATs, and HDAC2/6 and SIRT1 KDACs (Table 1). Upon a closer look, targets of p300-mediated acetylation are generally opposed by SIRT1-dependent deacetylation, while the targets of TIP60 may be opposed by HDACs but remain largely unknown. Following this train of thought, targets of p300/SIRT are activated by the loss of acetylation, whereas it is the addition of acetyl group to TIP60 targets that triggers their activation (summarized in Table 1, shown in Fig. 2).

Autophagy is stimulated by the depletion of key nutrients including amino acids, growth factors and glucose. Recognition of nutrient availability by multiple intracellular sensors converges on a handful of regulators that integrate nutrient signals into several key responses. These include mammalian target of rapamycin complex 1 (mTORC1) (Rabanal-Ruiz et al. 2017), glycogen synthase kinase 3 (GSK3) (Mancinelli et al. 2017), and adenine monophosphate-activated protein kinase (AMPK) (Tamargo-Gómez and Mariño 2018). Perhaps unsurprisingly, these three kinases have also been directly linked to the regulation of KATs and KDACs that influence the acetylation status of autophagy proteins. mTORC1 was recently shown to activate the acetyl-transferase activity of p300 by serine phosphorylation that was lost upon amino acid starvation (Wan et al. 2017). Activation of GSK3β by the loss of growth factor signalling is known to phosphorylate and thus activate TIP60 (Lin et al. 2012). Finally, AMPK activation releases SIRT1 inhibition in a GAPDH-dependent manner in response to glucose starvation (Chang et al. 2015). Thus, three potential axes regulate autophagy stimulation in response to nutrient stress by (a) loss of FoxO, VPS34, Beclin1, ATG7, ATG5 and ATG12 acetylation (amino acids/growth factors-mTORC1-p300), (b) increased ULK1 and possible ATG3 and p62 acetylation (serum/ER stress-GSK3β-TIP60) (Lin et al. 2012; Nie et al. 2016; Yi et al. 2012; You et al. 2019a), and (c) FoxO, Beclin1, ATG7, ATG5 and ATG12 deacetylation (glucose–AMPK–GAPDH–SIRT1) that could explain the conundrum of the varied nature of autophagy protein acetylation status upon nutrient starvation and its link to autophagy stimulation.

## Targets of cysteine oxidative PTMs in autophagy

Protein modification by ROS and RNS constitutes a covalent modification of amino acid residues by the reactive species directly, or as a secondary interaction in an oxidative relay. Briefly, irreversible (carbonylation, nitration) oxidative modifications affect a variety of amino acids including cysteine (Cys), threonine and tyrosine (Ahmad et al. 2017; Cai and Yan 2013; Xie et al. 2018). In contrast, reversible amino acid oxidation involves modification of the thiol group (-SH) of Cys protein residues that are first modified to sulfenic acid (–SOH) (Cai and Yan 2013). Sulfenic acid can then undergo nitrosylation (-SNO) by reacting with RNS, or disulphide bond formation (R–S–S–R) by intra-/inter-molecular bond formation between two cysteine residues. A specialised form of disulphide bond formation, glutathionylation (R–S–S–G) arises as a mixed disulphide bond formation between a target protein Cys residue and the non-enzymatic antioxidant, glutathione (GSH) (Cai and Yan 2013). Further oxidation of –SOH results in an irreversible Cys oxidation by the formation of sulfinic (–SO_2_H) and sulfonic (–SO_3_H) acids (Ahmad et al. 2017; Cai and Yan 2013; Murray and Van Eyk 2012). Autophagy regulation by ROS is linked to the reversible oxidative Cys modification of (a) transcription factors (TFs) that regulate expression of proteins involved in the autophagy process, (b) upstream regulators of autophagy initiation, (c) autophagy proteins themselves and (d) receptors that mediate autophagy substrate selectivity (Filomeni et al. 2015; Montagna et al. 2016; Sedlackova et al. 2020).

The most substantial link between ROS and autophagy TF activation was established in the studies of the Mit/TFE family of transcription factors (Yang et al. 2018). Three members of the Mit/TFE protein family were recently shown to contain redox sensitive Cys residues (TFEB Cys^212^, TFE3 Cys^322^, MITF Cys^281^) that mediate a rapid response to increased intracellular oxidative stress by promoting their nuclear translocation (Wang et al. 2019a). Another layer of regulation by oxidative stress was previously uncovered for TFEB that regulates expression of several autophagy proteins including, ATG4, ATG9, LC3B and p62 (Settembre et al. 2011). Increased intracellular oxidative stress is sensed by the lysosomal cation channel, mucolipin 1 (MCOLN1/TRPML1) in a manner that is not yet understood (Zhang et al. 2016c). What is known is that MCOLN1 oxidation promotes channel opening, Ca^2+^ release from the lysosomal lumen and activation of a Ca^2+^ dependent phosphatase, calcineurin (Medina et al. 2015; Zhang et al. 2016c). Calcineurin-dependent TFEB phosphorylation then promotes TFEB translocation to the nucleus and autophagy stimulation (Fig. 2A).

At the stage of autophagy execution, redox-sensitive Cys residues were identified in proteins involved in LC3 processing (ATG4B) and LC3-PE conjugation (ATG7 and ATG3). ATG4B is a Cys-dependent protease that cleaves pro-LC3 at a C-terminal glycine residue prior to LC3-PE conjugation (Kirisako et al. 2000). Its protease activity is also involved in correcting the amount of LC3–PE formation on non-autophagic membranes by the hydrolysis of the LC3–PE bond, and presumably on the outer membrane leaflet of the growing autophagosome. In human cells, the hydrolysing (deconjugating) activity of ATG4B is inhibited by the oxidation of one of two Cys residues (Cys^74^ or Cys^78^) and leads to improved stability of LC3–PE and increased formation of autophagosomes (Scherz‐Shouval et al. 2007). Similarly, oxidation of the catalytic thiols in ATG3 (Cys^264^) and ATG7 (Cys^572^) inhibits their activity in LC3–PE conjugation and results in the loss of autophagic flux (Fig. 2b) (Frudd et al. 2018). Interestingly, oxidation of these Cys residues can only occur when the thiols are not shielded by their interaction with LC3.

Oxidative stress influences the selectivity of the autophagic process via p62, a known redox sensitive autophagy receptor protein (Fig. 2c). Intermolecular disulphide formation in p62 was first observed in studies of its involvement in the N-end rule pathway of substrate degradation, where Cys^113^-dependent oligomerisation promoted substrate clearance via autophagy (Cha-Molstad et al. 2017). Subsequently, we have demonstrated that elevated ROS levels promote the formation of disulphide-linked conjugates, intermolecular Cys bonds, that assist p62 oligomer assembly (Carroll et al. 2018). Crucially, we have identified two Cys residues (Cys^105^ and Cys^113^) located within the regulatory linker region of the p62 protein, that are necessary and sufficient for the activation of pro-survival autophagy triggered by increased ROS (Carroll et al. 2018).

Reversible oxidation of Cys residues in redox-sensitive autophagy proteins thus appears to have a dual role of pathway stimulation by autophagy gene expression (TFEB), increased autophagosome formation (ATG4B) and substrate selectivity (p62), and autophagy inhibition upon depletion of available LC3 substrate (ATG3, ATG7). However, due to the novelty of these findings, the physiological role of ATG3 and ATG7 inhibition and possible downstream signalling events remain unknown. We propose a regulatory feedback loop whereby sensing depletion of local LC3 pools results in inactivation of ATG3 and ATG7 that serves to prevent indiscriminate autophagy activation. We envision that this inactivation would persist until such a time that the antioxidant defences decrease the oxidative stress load and resolve the ATG3-ATG7 heterodimer, and the expression of autophagy genes restores the available pools of ATG proteins to sustain further autophagy.

## The interrelatedness of target oxidation and acetylation in autophagy

Protein deacetylation and oxidation appear to be individually sufficient to regulate the initiation, promotion, efficiency and selectivity of autophagy. However, an interesting crosstalk between oxidative and acetyl-linked PTMs of autophagy proteins arises due to the dual control of several proteins including TFEB, ATG3, ATG7 and p62, which appear to be regulated by both, oxidation and acetylation status (Fig. 2a–c). First, upstream oxidation of MCOLN1 regulates TFEB localization by calcineurin-dependent dephosphorylation (Medina et al. 2015; Zhang et al. 2016c) and direct oxidation of its Cys^212^ residue (Wang et al. 2019a). Further, TFEB deacetylation at residues Lys^274^ and Lys^279^ promotes its dimerization and increases its binding affinity for DNA (Wang et al. 2019b). It would be interesting to study whether oxidation and acetylation PTMs act in concert to establish the optimal TFEB activity and whether TFEB oxidation promotes rapid expression of its target genes in the absence of Lys residue deacetylation.

Second, ATG3 acetylation at residues Lys^19^ and Lys^48^ by TIP60, increased in conditions of nutrient starvation, improves interaction between ATG3 and LC3 and promotes autophagy (Yi et al. 2012). Not much is known regarding the functional effect of deacetylation in ATG7, except that it promotes autophagy and Lys^306^ residue may be the target (Choudhary et al. 2009). In contrast to TFEB, a recently published study suggests that upon loss of LC3 binding, oxidation of ATG3 (Cys^264^) and ATG7 (Cys^572^) catalytic cysteine residues inhibits their enzymatic activity and blocks their further interaction with LC3 (Frudd et al. 2018).

Lastly, oxidation and acetylation of p62 could act in concert to achieve optimal selectivity of its interaction with cargo and oligomerization to stimulate autophagy. First, TIP60-dependent acetylation of Lys^420^ and Lys^435^ within the UBA domain interferes with its inter-protein dimerization and enhances the ubiquitin binding affinity of p62 upon serum and amino acid starvation (You et al. 2019a). Second, oxidation of Cys^105^ and Cys^113^ residues within the regulatory linker region promotes intermolecular p62 disulphide bond formation and thus assist in autophagy stimulation (Carroll et al. 2018).

In addition, activity of NAD^+^-dependent KDACs, or SIRTs, is directly or indirectly regulated by both, oxidative and metabolic stress stimuli. First, a shift in the NAD redox balance towards oxidation, suggestive of metabolic stress, leads to an increased pool of available NAD^+^ and thus stimulates SIRT activity (Imai and Guarente 2016). Second, SIRT regulation by oxidative stress was demonstrated in multiple cell culture experiments (reviewed in (Santos et al. 2016)), in which a mild oxidative environment promotes SIRT1 expression and activation by upstream kinases. In contrast, study of SIRT1 oxidation, specifically nitrosylation (–SNO^+^), suggests that this reversible oxidative PTM of Cys^371^, Cys^374^, Cys^395^ and Cys^398^ residues within a tetrathiolate formation results in loss of Zn^2+^ binding, structural destabilization and loss of NAD^+^ and acetyl-lysine binding ability (Kalous et al. 2016). Thus, SIRT1 activity can be stimulated by both, nutrient starvation, and oxidative stress. However, persistent ROS release may lead to SIRT1 destabilization, loss of its deacetylase activity and might contribute to its degradation by the proteasome (Caito et al. 2010).

## NAD depletion, oxidative stress, and autophagy in physiological and pathological ageing

The NAD nucleotide is an important redox molecule required for fundamental molecular processes of energy generation via glycolysis, tricarboxylic acid cycle, oxidative phosphorylation and β-oxidation, and a co-factor to enzymes involved in cellular signalling and longevity. Age-related depletion of available NAD^+^ pools was, in human disease, animal models and in vitro studies, reported as a result of increased PARP activity due to an elevation in oxidative stress and levels of DNA damage (Pacher and Szabo 2008) and increased CD38 expression and activity (Camacho-Pereira et al. 2016; Polzonetti et al. 2012). Combined with the age-dependent reduction in the enzymatic activity of nicotinamide phosphoribosyltransferase (NAMPT), the rate-limiting enzyme of the NAM-based NAD^+^ salvage pathway (Stein and Imai 2014), these conditions perpetuate the perfect storm of total NAD depletion, loss of NAM recycling and reduction in SIRT activity in physiological ageing.

Study of human skin tissue from volunteers of different ages partially supports these findings (Massudi et al. 2012). In this study, an age-dependent increase in DNA damage correlated with an increase in PARP activity, NAD^+^ depletion and, in the elderly, a reduction in SIRT1 activity. Notably, these associations with age were strong only in the male participants and it would be interesting to see whether these findings can be reproduced in females and other accessible human tissues, including muscle or post-mortem brain tissues. In a more recent study carried out on human skeletal muscle samples, authors demonstrate that levels of NAMPT negatively correlate with age, body mass index and body fat percentage (de Guia et al. 2019). Another study utilised the power of magnetic resonance-based non-invasive in vivo imaging of the human brain and revealed an age-dependent decrease in total NAD levels, concomitant with an increase in NADH/NAD^+^ ratio, indicative of metabolic dysfunction (Zhu et al. 2015). While these studies were carried out on healthy human volunteers and suggest that a decline in NAD levels occurs in physiological ageing, multiple studies of accelerated human ageing (progeria) syndromes and patients suffering from metabolic and neurodegenerative diseases strongly link NAD decline to age-related pathology (Kubben and Misteli 2017; Lautrup et al. 2019; Okabe et al. 2019).

Furthermore, studies of two age-related conditions, sarcopenia and frailty, as well as a variety of progeria, neurodegenerative, metabolic and cardiac diseases, demonstrate a strong link between pathology and increased oxidative stress (Derbré et al. 2014; Inglés et al. 2014; Kubben and Misteli 2017; Liguori et al. 2018; Massudi et al. 2012; Soysal et al. 2017). Not only does lipid peroxidation, a proxy measurement for increased oxidative stress, correlate with age (Massudi et al. 2012), a systematic review of available literature suggests that long lived humans (centenarians) have lower levels of oxidative protein damage and lipid peroxidation compared to other elderly individuals (Belenguer-Varea et al. 2019). Given the number and severity of clinical conditions related to healthy ageing, and age-related diseases that are associated with an increase in oxidative stress, it is necessary to design interventions that prevent production of free radicals, boost cellular antioxidant systems, or understand and target the processes downstream of ROS-mediated protein, lipid or nucleotide damage.

Importantly, molecular studies of free radical generation, NAD^+^-dependent enzymatic processes and disease pathology suggest a link between ROS accumulation, NAD depletion and compromised mitochondrial recycling by autophagy, mitophagy. Mitochondria are energy-generating organelles that act as hubs of pro-survival or pro-apoptotic signalling (Sedlackova and Korolchuk 2019). Although mitochondrial health is maintained by a complex net of quality control mechanisms, whole organelle recycling of damaged and ROS-producing mitochondria is only achieved by selective autophagy. A causal link between NAD^+^ depletion and mitochondrial dysfunction due to loss of mitophagy was established in studies of premature ageing syndromes including Xeroderma Pigmentosum, Cockayne syndrome and Ataxia-telangiectasia (Fang et al. 2016, 2014; Scheibye-Knudsen et al. 2014, 2012; Valentin-Vega et al. 2012). In these studies, loss of SIRT activity and autophagy abnormalities occur as a result of PARP1 hyperactivation due to unresolved DNA damage. In addition to SIRT inactivation, uncontrolled NAD^+^ cleavage and protein PAR-ylation by PARPs results in loss of ATP availability and, if persistent, in cell death (Andrabi et al. 2014; Bai et al. 2011; Fouquerel et al. 2014; Pillai et al. 2005). Persistent NAD^+^ depletion was thus shown to compromise mitochondrial function due to loss of energy generation, impairment in mitochondrial recycling through lack of autophagy/mitophagy stimulation, and to initiate cellular death due to energy collapse. An alternative outcome to cell death upon PARP1 activation was linked to autophagy initiation in independent cell culture experiments (Jiang et al. 2018; Muñoz-Gámez et al. 2009). In the earlier study, authors demonstrated that PARP-dependent stimulation of autophagy due to short-lived energy crisis had a cytoprotective effect as genetic or pharmacological inhibition of autophagy led to increased level of necrotic death (Muñoz-Gámez et al. 2009). In the latter study, authors aimed to mimic constant ROS production in vivo by glucose oxidase (GO) treatment, which led to PARP-induced cell death, parthanatos (Jiang et al. 2018). In this study, inhibition of autophagy led to a significant collapse in mitochondrial polarization and an approximately 50% increase in cell death within four hours of GO treatment. Taken together with the role of SIRT-mediated autophagy stimulation, we wonder whether convergence of these signalling pathways on autophagy suggests a conserved role of this catabolic pathway in healthy ageing by preservation of cellular NAD pools.

An exciting development in the field of ageing and NAD metabolism is the ‘druggability’ of NAD metabolism by exogenous addition of natural, or synthetic, bioavailable NAD^+^ precursors. This universal approach of NAD^+^ precursor supplementation is known to increase NAD biosynthesis and alleviate the symptoms of pathological states including metabolic, cardiac and neurodegenerative disorders (Kane and Sinclair 2018; Lautrup et al. 2019). Additionally, evidence from NAD^+^ supplementation studies in cell culture and in animal models suggests that boosting NAD levels is sufficient to not only improve mitochondrial function, but also stimulate SIRT-dependent mitochondrial recycling via increased TFEB- and FoxO-dependent expression of autophagy/mitophagy genes and PTMs of autophagy proteins, and thus promote clearance of dysfunctional organelles and protein aggregates (Fang et al. 2019, 2016; Hou et al. 2018; Schöndorf et al. 2018; Vannini et al. 2019; Zhang et al. 2016b). Altogether, this ‘silver bullet’ approach might serve as an intervention to the vicious cycle of damage and NAD depletion and thus not only combat the depletion itself, but also support resolution of the underlying stresses and promote long-term cellular health.

Following the success of NAD^+^-boosting strategies in cell and animal models, NAD^+^ precursors, and predominantly nicotinamide riboside (NR), are now subjects of multiple clinical trials. Precursors have so far been reported as safe, well tolerated and capable of increasing NAD levels in healthy volunteers (Conze et al. 2019; Martens et al. 2018; Minto et al. 2017; Stea et al. 2017). However, challenges remain in translation of laboratory findings into the design of clinical trials (Gilmour et al. 2020). While some early success was found in disease outcomes of amyotrophic lateral sclerosis (ALS) (NCT03489200) (de la Rubia et al. 2019), others found no benefit in patients with Alzheimer’s disease (NCT00580931) (Phelan et al. 2017), or studies of metabolic disorders or mitochondrial bioenergetics in men (NCT02303483) (Dollerup et al. 2018, 2019a, 2019b). Although only a limited number of trials testing NAM, NMN and NR have been recently completed (> 10) or are currently ongoing (− 3), many are actively recruiting (− 21) (https://clinicaltrials.gov/) (Lautrup et al. 2019) and it will be interesting to see what lessons can be learned about precursor dosage, NAD^+^/NADH detection methods and bioavailability in the coming years. Considering that the pathological role of NAD depletion in many metabolic and neurodegenerative diseases is not yet firmly established, reporting of relevant disease outcomes is eagerly awaited as they will inform about the feasibility of translating success from the laboratory to human age- and disease-related interventions.

## Concluding Remarks

Autophagy is a highly conserved catabolic process that is controlled by multiple nutritional and stress-related cues by reversible protein PTMs. In this review, we first explored the latest findings on how two PTMs, Lys acetylation and Cys oxidation, regulate the localization and function of autophagy proteins. Collectively, novel findings published in 2015–2020 identify TFEB, ULK1, VPS34, ATG3, LC3 and p62 as targets of acetylation PTMs which, in response to metabolic cues, stimulate the expression and enzymatic activity of autophagy proteins and improve pathway selectivity. Furthermore, Mit/TFE family of TFs (including TFEB), ATG3, ATG7 and p62 are also known to contain redox-sensitive Cys residues the oxidation of which influences autophagy outcomes. The dual control of protein localization/enzymatic activity by acetylation and oxidation links the efficiency of autophagy outcomes to nutrient loss and metabolic dysfunction and thus contributes to cellular homeostasis and healthy ageing.

Crucially, studies of the molecular mechanisms of NAD function in cellular physiology and ageing suggest a central role of autophagy in first, preventing increases in DNA damage and NAD^+^ consumption via mitochondrial recycling and second, by alleviating nutritional crisis via recycling amino acids, lipids and nucleosides. Autophagy thus appears to be necessary in supporting cellular survival upon either nutritional stress that changes NAD redox ratio towards the oxidised form (NAD^+^) and stimulates SIRTs, or upon DNA damage followed by NAD^+^ depletion due to PARP1 hyperactivation. Thus, although short term insults to cellular heath are resolved by autophagy stimulation and cellular detoxification, we wonder whether persistent oxidation and NAD^+^ loss in aged tissues result in stalled autophagy, and due to lack of stress resolution, ultimately in loss of cell viability and tissue dysfunction.
